# Described neural connections enhance classroom learning of neuroanatomy

**DOI:** 10.1002/ase.70051

**Published:** 2025-06-07

**Authors:** Nicholas C. Hindy, Anthony J. Bishara, John R. Pani

**Affiliations:** ^1^ Department of Psychology College of Charleston Charleston South Carolina USA; ^2^ Department of Psychological and Brain Sciences University of Louisville Louisville Kentucky USA

**Keywords:** computer‐aided instruction, connectivity, neuroanatomy, systems neuroscience, undergraduate education

## Abstract

Advances in brain imaging have led to a paradigm shift in neuroscience research, moving from focusing on individual brain structures to investigating neural networks and connections. However, neuroanatomy education still tends to concentrate on discrete brain regions. Two separate experiments in undergraduate neuroscience courses investigated whether incorporating neural connectivity into neuroanatomy education would enhance learning. Students in each experiment learned to identify brain structures through computer‐based training sessions that provided text‐based narrative feedback about neural connections, followed by final memory tests after a 1‐month delay. The first experiment included 30 students and demonstrated a long‐term memory benefit associated with described neural connections, showing a medium effect size (*p* = 0.01, *d* = 0.54) comparable to the established retrieval practice effect for enhancing long‐term memory (*p* = 0.03, *d* = 0.47). The second experiment replicated the benefits of described neural connections with a small effect size (*p* = 0.005, *d* = 0.28) in a larger sample of 122 students across classrooms at two universities. Furthermore, students remembered the functional outcomes of neural connections from training (*p* < 0.001, *d* = 0.46), and this generalized to clinical applications (*p* = 0.009, *d* = 0.27). In contrast, categorizing brain areas without describing neural connections (as is commonly done in introductory neuroscience textbook chapters) did not benefit either memory or generalization. Findings demonstrate that leveraging the connectivity paradigm shift in neuroscience research can enhance neuroanatomy education. Emphasizing neural connections and their functional outcomes helps simplify neuroanatomy and improve understanding and retention.

## INTRODUCTION

Neuroanatomy is foundational to the rising number of undergraduates enrolled in neuroscience courses,^1^ but students often view neuroanatomy as difficult, dry, and irrelevant.^2^, ^3^, ^4^, ^5^ Poor engagement leads to quick forgetting of neuroanatomy material.^6^, ^7^ Traditionally, the introduction of neuroanatomy in undergraduate neuroscience courses has been structured around discrete brain areas, often emphasizing their phylogenetic development or isolated functions.^8^, ^9^ While systematic, this approach does not reflect the growing body of research that underscores the structural and functional networks within the brain.^10^, ^11^

Recent advances in brain imaging and analysis have spurred a paradigm shift in neuroscience research from focusing on individual brain structures to investigating neural networks and connections.^12^ Despite its potential, the pedagogical advantages of using neural connectivity as a teaching tool in neuroscience education have yet to be investigated. We aim to improve neuroanatomy instruction in undergraduate STEM education by using neural connectivity to associate brain structures with each other and with functional outcomes.

### Neural connectivity in neuroscience research

Catalyzed by large‐scale initiatives, such as the Human Connectome Project,^13^, ^14^ much of neuroscience research has moved from studying individual brain structures to exploring their connections. Structural imaging methods, such as diffusion tensor imaging (DTI), reveal the white matter pathways that connect brain areas with one another and contribute to large networks.^15^, ^16^, ^17^, ^18^ At the same time, functional neuroimaging methods, such as resting‐state^19^, ^20^ and task‐based^21^, ^22^ functional connectivity, reveal how brain areas interact within these networks. Examples of large‐scale brain networks revealed by functional connectivity include the salience network involved in orienting to behaviorally relevant stimuli in the environment,^23^ the dorsal attention network involved in visuospatial attention,^24^ the frontoparietal network involved in cognitive control,^25^ and the sensorimotor network involved in coordinated motor tasks.^26^ These networks span traditional anatomical boundaries^27^ and provide valuable biomarkers for psychopathology.^28^ Neural connections and disconnections revealed by DTI and measures of functional connectivity predict long‐term symptoms in stroke patients,^29^ patient‐specific benefits of deep brain stimulation,^30^ and individual differences in abilities and behaviors.^31^, ^32^

### Neuroanatomy in undergraduate education

**FIGURE 1:**
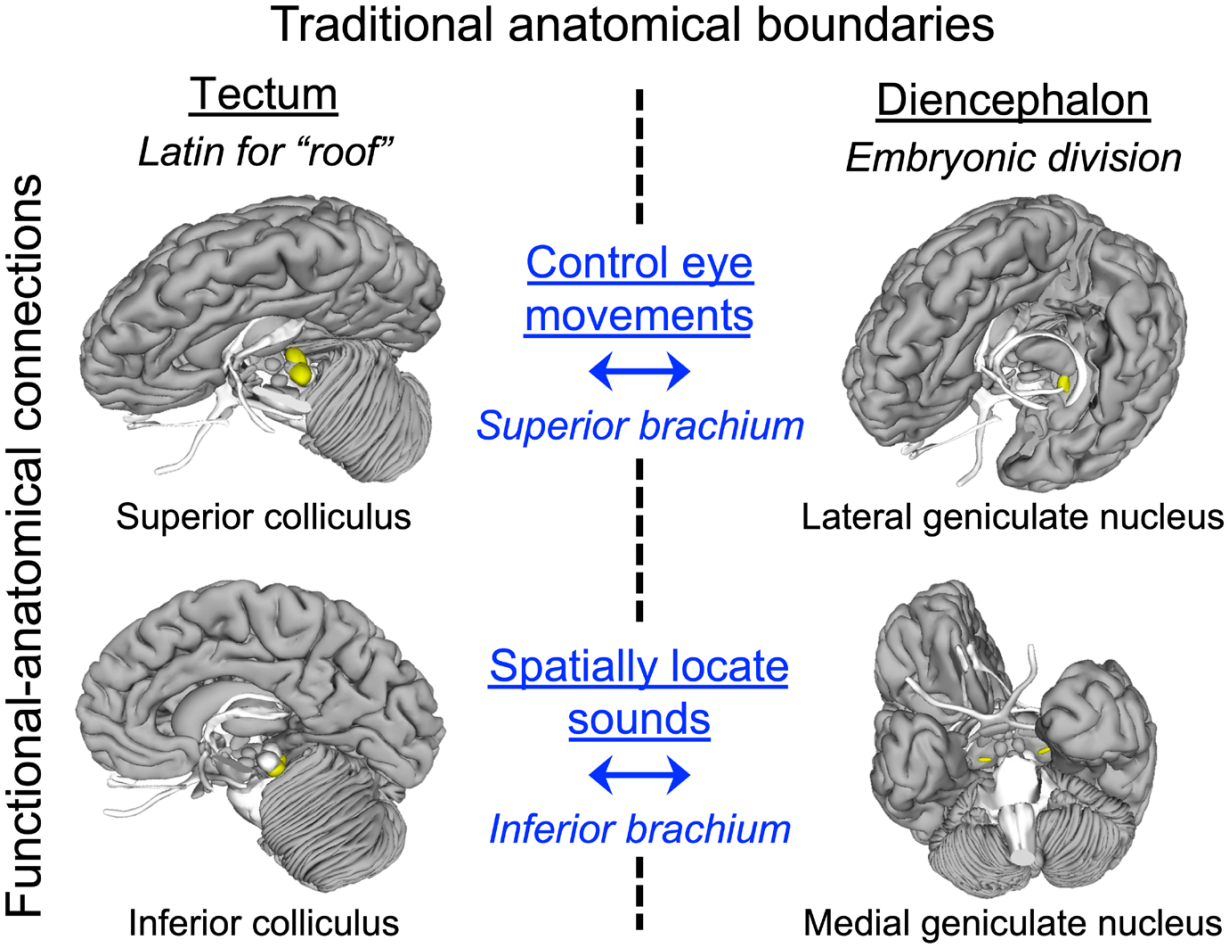
Functional–anatomical connections versus traditional anatomical boundaries. Associations based on neural connections can be more meaningful to undergraduates than traditional neuroanatomical boundaries.

While there have been innovations in teaching neuroanatomy, such as 3D modeling and virtual reality, these educational methods have not advanced as rapidly as brain imaging technologies in research and health care.^33^, ^34^ While later chapters of an introductory neuroscience textbook focus on brain functions and systems, early neuroanatomy sections of textbooks continue to emphasize traditional anatomical divisions often unrelated to structural or functional connectivity.^35^, ^36^, ^37^, ^38^ For example, the tectum includes the superior colliculus and inferior colliculus (Figure 1). However, the superior colliculus is part of the visual system, while the inferior colliculus is part of the auditory system. Divisions, such as “tectum,” are irrelevant to other textbook chapters that categorize experiments and clinical applications based on functional systems. In contrast to traditional anatomical boundaries, neural connectivity includes the anatomical connections among brain regions and the specific cognitive and behavioral functions that these connections serve. For instance, the superior brachium anatomically connects the superior colliculus and the lateral geniculate nucleus to control eye movements,^39^ and the inferior brachium connects the inferior colliculus and the medial geniculate nucleus to locate sounds spatially.^40^

Focusing on traditional anatomical boundaries may be counterproductive to undergraduate neuroanatomy education, as it emphasizes features unrelated to function. Highlighting such features may impede problem‐solving performance.^41^ For instance, novices tend to focus on surface similarity when classifying and trying to solve problems.^42^, ^43^ That is, they tend to focus on perceptually salient similarities, such as similarities that arise from (1) an idiosyncratic image, perhaps due to a particular angle; (2) similar shapes, such as the spherical shape of the inferior and superior colliculus; or (3) similar words (“colliculus”) or letter combinations (ending in “erior”). In contrast, experts tend to focus on features that connect to appropriate schemata.^44^, ^45^ Such schemata cue appropriate solutions to problems (e.g., If a patient has vision problems, which structures might be damaged?).

Focusing on neural connections in neuroanatomy instruction may implicitly help students create memory schemas by focusing on neural connections to provide critical relationships among to‐be‐learned materials.^46^ This approach aligns with a constructivist framework for education.^47^, ^48^ Providing relational contexts (spatial, causal, and functional associations) for new knowledge can facilitate the construction of coherent mental models, which are particularly challenging when learning about the complex and abstract relationships between brain structures. Emphasizing neural connectivity in neuroanatomy education can scaffold students' knowledge and make the content more manageable, meaningful, and memorable.

Causal relationships between neural connections and mental or behavioral outcomes may encourage a student to construct a rich mental model of the brain that connects spatial location, causes, and functional outcomes of different neural connections,^49^, ^50^ leading to a single integrated representation of neuroanatomy rather than many disconnected ones.^51^ Learning about neural connectivity may also help students scaffold new learning on existing knowledge.^52^ For example, most undergraduates are familiar with the cerebellum but not the red nucleus.^53^ If students learn that the red nucleus connects with the cerebellum to coordinate actions, they can remember it by associating an unfamiliar brain area with a familiar one. Even without explicitly teaching students how to construct these schemata, instruction focusing on neural connections in neuroanatomy instruction may enhance learning by providing critical relationships among to‐be‐learned materials.

### Computer‐based neuroanatomy learning

We used an interactive graphical software system called Show Me the Brain!! (SMtB) for each training session and the final memory tests^54^ (J.R. Pani Instructional Systems, USA). SMtB is a revised and expanded version of a system that has been thoroughly evaluated in the context of university instruction in various undergraduate courses and validated in several laboratory studies^55^, ^56^, ^57^, ^58^ and the classroom.^59^ It includes unprecedented accuracy in the three‐dimensional (3D) graphical representation of the human brain and depicts both cortical and subcortical brain structures. The 3D models are based on source material from the Visible Human 2.0 (VH2) cryosections^60^ (National Library of Medicine, USA). The images in SMtB consist of high‐resolution MRI scans, the VH2 cryosections, and the 3D computer models developed from the VH2 cryosections. Only the 3D computer models were used in the current experiments.

**FIGURE 2:**
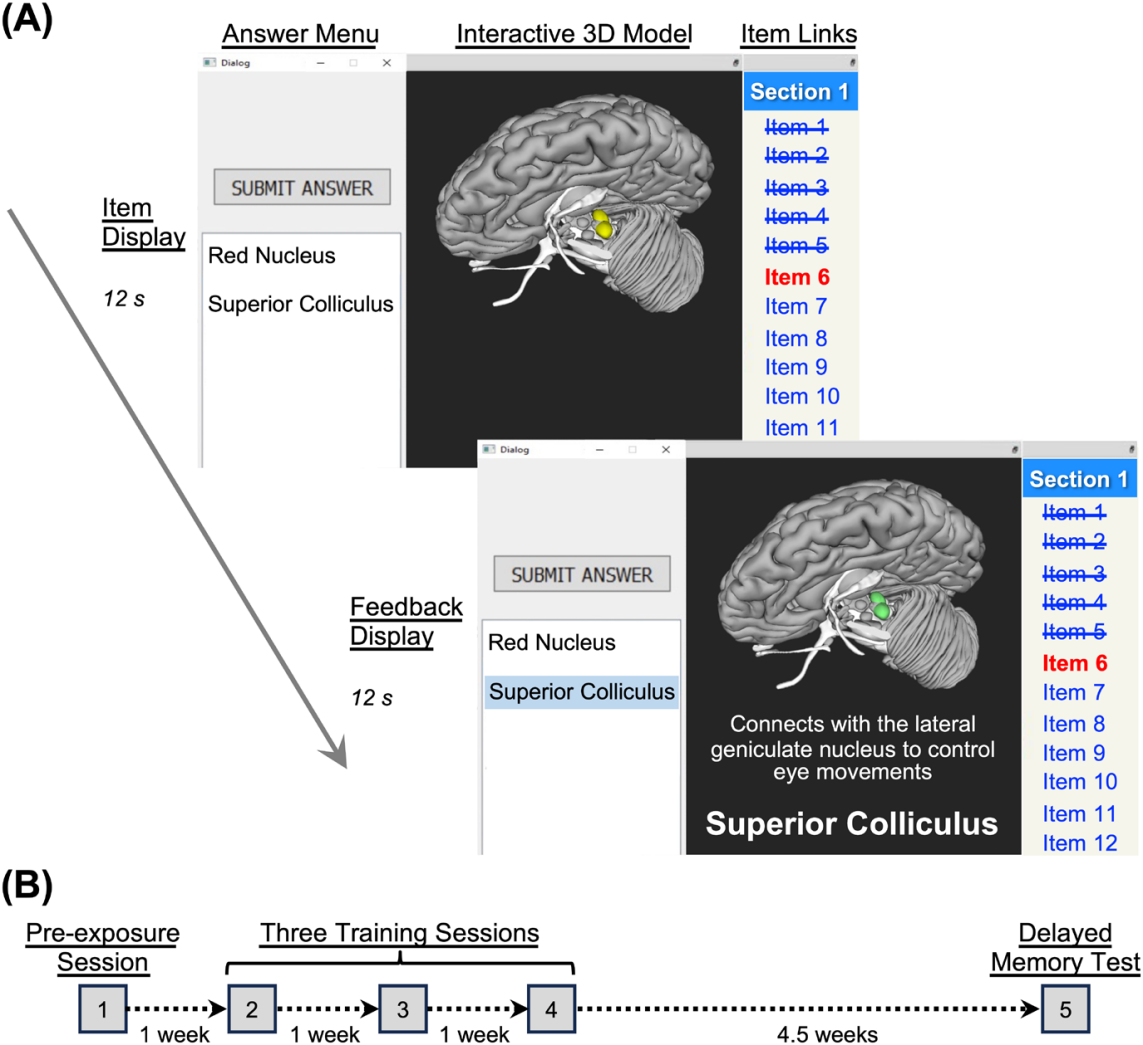
Show Me the Brain!! (SMtB) interface and experiment procedure. (A) SMtB includes an interactive 3D viewer, a window with HTML links, and an answer menu that appears in “Test Mode.” In each experiment, participants began each training trial by clicking an item link. The 3D model of the test item appeared, and participants clicked the brain structure label in the answer menu. After the 12‐s item display, the highlighted structure turned either green (correct) or red (incorrect). The correct label was displayed as feedback, and a described neural connection was presented above the structure name for structures in the “described connection” conditions. (B) We conducted each experiment as five sessions spaced across 7.5 weeks.

The SMtB software system includes an interactive 3D viewer and an HTML window (Figure 2A). When SMtB is in “Test Mode,” an answer menu also appears. The 3D model of the brain can be controlled by directly clicking and moving it around or by clicking HTML links in the same way one would control a web page. These links specify which brain areas are visible in the display, the initial viewpoint parameters of the brain, the available answer options, and feedback that can be displayed after a user answers a question.

### Retrieval practice for learning neuroanatomy

Learning is significantly enhanced when students engage in retrieval practice—actively recalling information from memory—rather than merely rehearsing or reviewing material presented to them.^61^, ^62^, ^63^ This cognitive strategy, also known as the testing effect, has proven to be a powerful learning tool across various areas of undergraduate education.^64^, ^65^ Retrieval practice may strengthen neural pathways and synaptic connections associated with the information, consolidating memory through Hebbian learning mechanisms (“neurons that fire together, wire together”).^66^, ^67^. This makes retrieval practice particularly effective for durable learning.^68^ By actively retrieving information, students improve long‐term memory retention and deepen their understanding more effectively than with other forms of active learning,^62^ especially when retrieval practice is spaced over extended periods.^69^

In medical education, retrieval practice has demonstrated significant benefits,^70^, ^71^ and its effectiveness extends specifically to neuroanatomy instruction.^72^ Recognizing its proven efficacy, retrieval practice is central to the classroom experiments described here and serves as a benchmark for evaluating the impact of connectivity‐based training on student learning outcomes.

### Introduction to experiments

Two experiments in undergraduate classrooms explored how emphasizing neural connectivity can enhance students' learning of neuroanatomy. We hypothesized that neuroanatomy instruction emphasizing connectivity would improve students' ability to associate brain structures with one another and their functions, leading to improved long‐term retention and generalization of neuroanatomical information. To test this hypothesis, the experiments involved within‐subjects designs in which the same students were exposed to multiple training conditions, with different brain structures assigned to each condition. In these experiments, students completed training sessions focused on identifying brain structures while receiving text‐based narrative feedback about neural connections. The first experiment compared the effects of described neural connections to retrieval practice effects on the long‐term memory of neuroanatomy. The second experiment aimed to replicate and extend the findings from the first experiment in a larger sample of undergraduates, a broader range of outcome measures to assess long‐term memory, and a new independent variable—anatomical categorization—to test whether simply grouping neuroanatomical content into functional systems (*linguistic*, *limbic*, *motor*, and *visual*) would support knowledge retention in the absence of described neural connections.

## MATERIALS AND METHODS

Experiments 1 and 2 employed identical materials and training procedures but differed in participants, secondary training manipulations (retrieval practice or categorization), and the scope of the final memory tests. We outline the general training procedure and differentiate between Experiment 1 and Experiment 2 as necessary.

### Participants

#### Experiment 1

Thirty undergraduate students at the College of Charleston participated in the experiment as part of a behavioral neuroscience course in September and October of 2021. Most students were in their second or third year majoring in psychology. Students participated in the experiment for course credit, and the number of students in the course determined the sample size. For all analyses, we included only students who completed all three training sessions and the final memory test, resulting in a final sample size of 25 participants (20 female and five male). The College of Charleston Institutional Review Board approved the study protocol (#2021‐08‐007).

#### Experiment 2

A total of 122 undergraduates participated in the experiment (29 students from the College of Charleston and 93 students from the University of Louisville) as part of introductory neuroscience courses in September and October of 2022. Students at each institution participated in the experiment for course credit, with the sample size determined by the number of instructors who volunteered to participate in the study and the number of students in the classes. Most students at each institution were in their second or third year majoring in psychology. For all analyses, we included only students who completed all three training sessions and all six memory tests. This resulted in a final sample size of 104 participants. The final sample included 24 participants from the College of Charleston (20 female and 4 male) and 80 participants from the University of Louisville (57 female and 23 male). The study protocol was approved by institutional review boards at both the College of Charleston (#2021‐08‐007) and the University of Louisville (#714502).

### Materials

The Show Me the Brain!! (SMtB) interactive graphical software system (J.R. Pani Instructional Systems, USA) was used for the pre‐exposure session, all training sessions, and several of the final memory tests. SMtB will be freely available as an open‐source package on GitHub. Students in each experiment accessed SMtB using their personal computers at home or on campus. Students at the College of Charleston used Amazon AppStream 2.0 (Amazon Web Services, Inc., USA)^73^ to connect to SMtB. Students at the University of Louisville connected to SMtB through Microsoft Azure Labs (Microsoft Corporation, USA).^74^ Both AppStream 2.0 and Azure Labs permit running a computer application from the internet cloud as if installed on the user's computer.

Stimuli included 3D graphical models of 48 brain structures and text‐based narrative feedback about labels, neural connections, and functional outcomes. Participants were trained and tested on associating the label of a brain structure with its graphical model. In the “described connection” conditions, students also learned about connected brain structures and the functional outcomes of neural connections. Table 1 lists examples of the brain structures and their described neural connections. Table S1 in the Supplemental Material lists all the brain structures and their described neural connections.

**TABLE 1:** Example brain structures and described neural connections.

Category	Brain structure	Described neural connection
Visual	Fusiform gyrus	Connects with the inferior temporal gyrus to recognize objects
	Inferior temporal gyrus	Connects with the fusiform gyrus to recognize objects
	Superior colliculus	Connects with the lateral geniculate nucleus to control eye movements
	Lateral geniculate nucleus	Connects with the superior colliculus to control eye movements
Linguistic	Middle frontal gyrus	Connects with Brodmann area 47 to retrieve words
	Brodmann area 47	Connects with the middle frontal gyrus to retrieve words
	Inferior colliculus	Connects with the medial geniculate nucleus to localize sounds
	Medial geniculate nucleus	Connects with the inferior colliculus to localize sounds

*Note*: Described neural connections were shown as text‐based narrative feedback after training trials. In Experiment 1, we selected brain structures within each condition from multiple categories (visual, linguistic, limbic, and motor). In Experiment 2, brain structures were either drawn from multiple anatomical categories (“uncategorized” conditions) or a single category (“categorized” conditions). Table S1 in the Supplemental Material includes all 48 brain structures and the corresponding described neural connections.

### Experiment timeline

Each experiment spanned five sessions across 7.5 weeks, including a pre‐exposure session, three training sessions, and a final memory test (Figure 2B). The timeline of events in training and testing was informed by previous studies on spaced retrieval practice.^70^, ^72^, ^75^ Participants were allowed a 24‐h time window to complete each session from their personal computers. The training procedures for Experiments 1 and 2 were identical, with the exception of the training manipulations described below and that Experiment 2 involved multiple‐choice with four response options instead of two response options.

Students did not have access to SMtB outside the designated sessions, and the memory tests were graded based on completion rather than accuracy to deter students from independently studying the brain structures by other means. More importantly, each experiment involved a within‐subjects design in which every participant experienced every condition, with the assignment of brain structures to conditions counterbalanced across participants. Students studying outside the neuroanatomy training sessions may have led us to underestimate differences among the training conditions. However, critically, differences among students studying outside the training sessions should not have affected one condition differently from any other.

### Pre‐exposure session

In the pre‐exposure session, the names of all 48 brain structures appeared as links on the right side of the screen and as answer options on the left side. Participants were not tested on the names of the brain structures during this session. Instead, they clicked the link for each structure name to display an interactive 3D brain model. One structure was highlighted in yellow, and the surrounding non‐highlighted structures provided a visual scene. The name of the highlighted structure appeared below the brain model. Participants selected the structure name from an answer menu containing all 48 structures and clicked a “Submit Answer” button for each brain structure.

### Training sessions

Each of the three subsequent training sessions comprised 96 trials, including two trials for each of the 48 brain structures. The trials within a training session were organized into eight sections of 12 trials each, allowing students the flexibility to complete the laboratory at their own pace within the 24‐h open window. Because of the fixed trial duration and number of trials for each training session, stimulus exposure was exactly 28.8 min for each condition across the three training sessions. However, students could take as much time as needed between the 96 trials and eight sections of each training session. The median total time to complete each training session was about 60 min, with an interquartile range of 50 to 75 min.

Each trial lasted 24 s. First, a highlighted brain structure appeared with its surrounding structures for 12 s, while alternative structure labels appeared as options in an answer menu to the left of the brain 3D model. These answer options included two different structure labels for each training trial in Experiment 1 and four different labels for each trial in Experiment 2. To prevent students from guessing the correct answers, foils in each experiment were randomly selected from a group of 15–17 similar structures for each correct structure label (Table S2 in the Supplemental Material). Participants clicked the structure label that matched the highlighted structure in the 3D model and then clicked a “Submit Answer” button. After the 12‐s item display, the highlighted structure turned either green (correct) or red (incorrect), and the correct name of the brain structure appeared as feedback for 12 s. Depending on the training condition, the structure name appeared by itself during the feedback display or with a described neural connection appearing below it. The initial camera angle was the same for each repetition of a particular brain structure, but students could rotate the brain model and view it from multiple angles. Figure 2A depicts an example section containing 12 item links.

### Manipulation of described neural connections

Both experiments included described neural connections as an independent variable. In the “described connection” conditions, the correct label of the structure appeared during the feedback part of a trial alongside a brief description of a neural connection with another brain structure and the functional outcome of this connection (e.g., “Connects with the lateral geniculate nucleus to control eye movements”). In the “label‐only” conditions, the correct label of the displayed brain structure appeared in training trials with no other information. After a one‐month delay for each experiment, we evaluated students' long‐term memory of the 48 brain structures based on structure identification in each experiment. The second experiment included a broader range of outcome measures to assess long‐term memory, including visual transfer and clinical applications.

### Experiment‐specific training manipulations

Training for each experiment involved an orthogonal second manipulation within a 2 × 2 factorial design, but differed with respect to the secondary training manipulation. Experiment 1 included retrieval practice as a secondary training manipulation, while Experiment 2 included anatomical categorization as a secondary training manipulation.

#### Experiment 1

**FIGURE 3:**
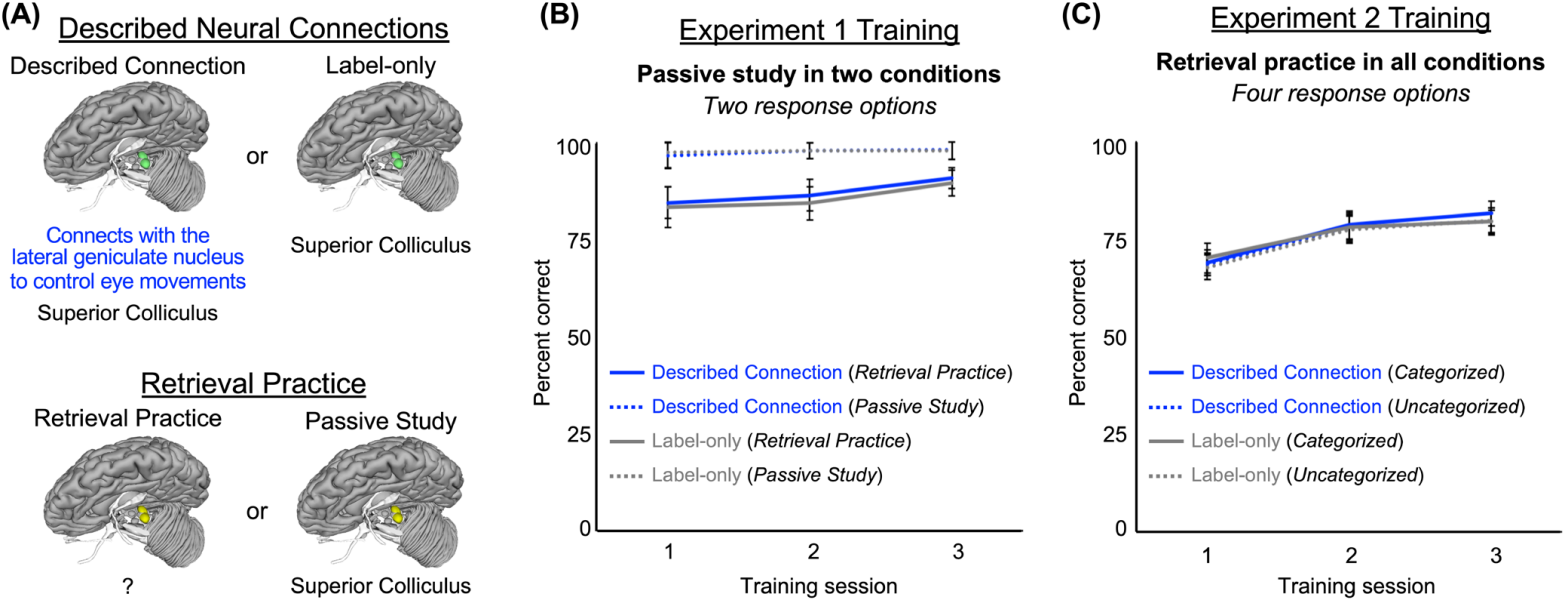
Training conditions and performance. (A) We manipulated the described neural connections in each experiment through text‐based narrative feedback displayed at the end of each trial in both experiments. In Experiment 1, we varied retrieval practice by either including or omitting the name of the brain structure at the beginning of each trial. All conditions in Experiment 2 involved retrieval practice. (B) In Experiment 1, training accuracy reached a ceiling for the passive study conditions. For the retrieval practice conditions, we observed a significant improvement in accuracy across the three training sessions. In contrast, retrieval practice accuracy showed no differences or interactions with the described connection and label‐only conditions. (C) Training accuracy in Experiment 2 improved across sessions, but no other significant main effects or interactions were noted.

Two types of item display distinguished “retrieval practice” conditions from “passive study” conditions (Figure 3B). In the retrieval practice conditions, the brain model appeared by itself for 12 s while the participant selected the label of the highlighted brain structure from two options. After 12 s, the structure label (and a described neural connection in half the trials) appeared for 12 s while the highlighted brain structure remained visible. Passive study trials were identical in procedure to the retrieval practice trials, except that the structure label appeared below the 3D brain model in both the first and last 12‐s periods. Additionally, passive study trials were randomly intermixed with retrieval practice trials to ensure student participants remained engaged throughout the training.

#### Experiment 2

To manipulate categorization, we compared training in which functionally related structures and connections were learned contiguously and in the context of a category label to when the structures and their connections were intermixed and uncategorized. In “categorized” conditions, brain structures and neural connections belonging to a single category (visual, motor, limbic, or linguistic; see Table 1 and Table S1 in the Supplemental Material) were always practiced within the same training sections, while the category label (e.g., “Visual System”) appeared at the top of the computer screen. In “uncategorized” conditions, brain structures and neural connections from two different categories (e.g., visual and motor) were intermixed within each training section, and “Miscellaneous Structures” was displayed at the top of the computer screen. All training trials in Experiment 2 used the retrieval practice method used in Experiment 1.

### Final memory tests

#### Experiment 1

Each participant completed a final memory test 4.5 weeks after the last training session. The final memory test was completed within SMtB, and brain structures appeared in the same orientation and with the same surrounding structures as during the training sessions. In the test, numbered item links (e.g., “Item 1”) randomly corresponded to each of the 48 brain structures. Participants clicked each link to display a 3D model of the highlighted brain structure and surrounding structures. Participants identified each brain structure by clicking its name within an answer menu that included the names of all 48 brain structures as options. No feedback was given. Each time participants clicked “Submit Answer,” the 3D model returned to its original state without any structures highlighted.

#### Experiment 2

Instead of one final memory test, Experiment 2 included six memory tests (Figure 5A). Tests were completed on personal computers within the same 24‐h time window as one another and in a fixed order (i.e., we tested all structures in Test 1 and then all in Test 2) to minimize contamination effects from earlier tests to later tests. Student participants performed Tests 1 and 2 within SMtB with brain structures appearing in the same orientation and with the same surrounding structures as during the training sessions. In Test 1 (cued recall), participants viewed each 3D brain structure and labeled it by typing its name into an answer box. Student responses could be in upper or lower‐case letters. Incorrect spellings were allowed if the Levenshtein distance was two or fewer single‐character edits,^76^ and it contained the correct number in the case of Brodmann areas (e.g., “Brodmann Area 45”). Test 2 (multiple choice) was identical to the final memory test in Experiment 1.

**FIGURE 4:**
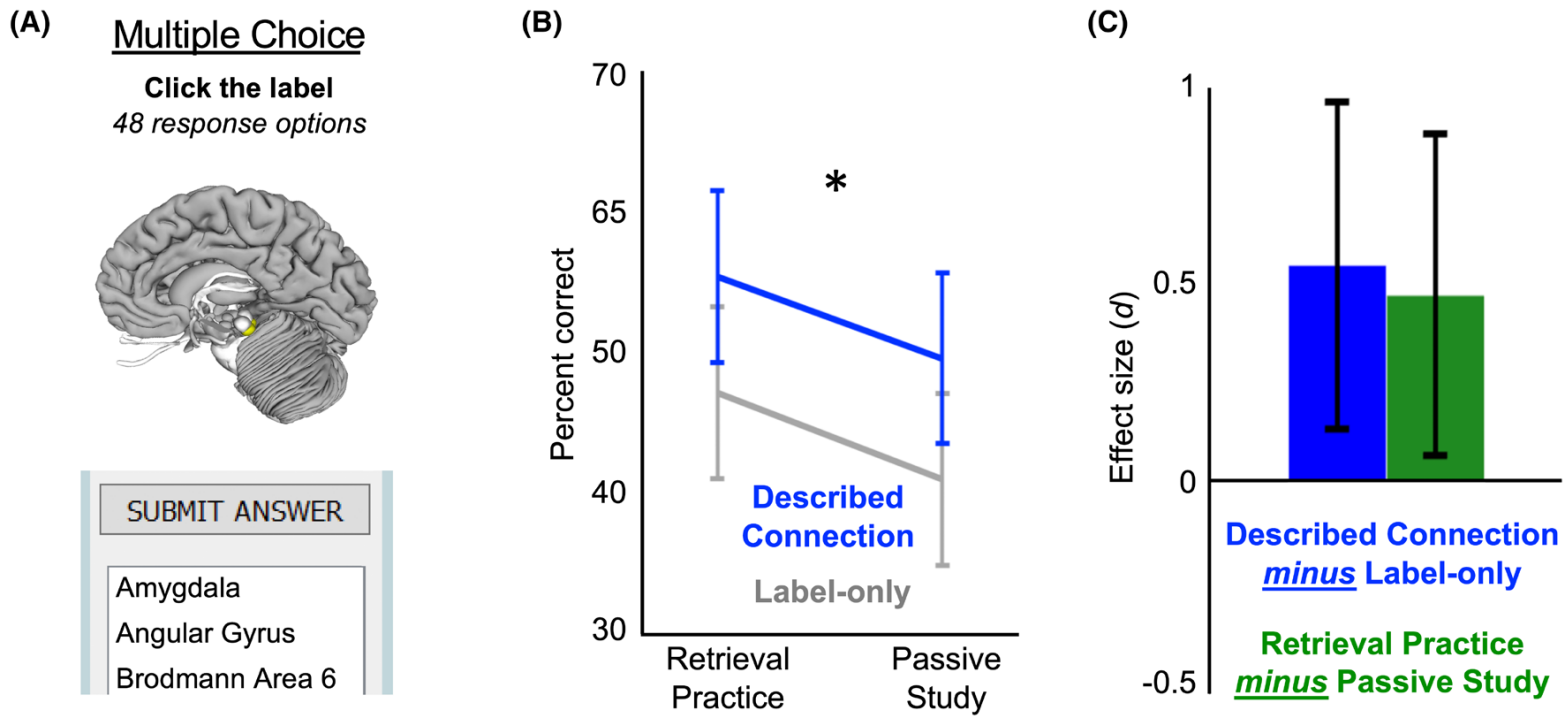
Experiment 1 long‐term memory. (A) Long‐term memory for each brain area was assessed using a multiple‐choice test with 48 response options. (B) We observed significant advantages in long‐term memory associated with both the described neural connections and retrieval practice. 95% confidence intervals and the significance marker reflect the main effect difference between the described connection and label‐only conditions. (C) Effect sizes for the two training manipulations. Error bars display the 95% confidence interval for each effect size. *p < 0.05.

Students completed Tests 3 to 6 outside of SMtB in Qualtrics (Qualtrics International Inc., USA) with screenshots taken from SMtB. In Test 3 (near visual transfer), participants viewed each 3D brain structure from a different camera angle and with different surrounding brain areas than in the training sessions. They labeled it by clicking within a menu with four alternative labels as options. In Test 4 (far visual transfer), participants viewed a 2D cryosection with the target brain structure highlighted and labeled the structure by clicking within a menu with four alternative labels as options. In Test 5 (functional outcomes), participants identified the function of each brain structure by clicking within a menu that included four alternative functions as options. For example, participants indicated “spatially locate sounds” for “Inferior Colliculus + Medial Geniculate Nucleus.” In Test 6 (clinical applications), participants read a scenario for each trial and then clicked which of four neural connections was most likely damaged within a menu with four alternative neural connections as options. For example, participants indicated “Transverse Temporal Gyrus + Superior Temporal Gyrus” for the clinical scenario, “After a stroke, Elizabeth can no longer distinguish between musical tones.” We measured performance for each test as percent correct.

### Data analysis

Data from the final memory tests and the training sessions were analyzed through repeated‐measures ANOVAs implemented in MATLAB (The MathWorks, Inc., USA).^77^ Tests were evaluated against a two‐tailed *p* < 0.05 level of significance, and effect sizes are reported as Cohen's *d* for repeated measures.^78^ Trials without a response (approximately 3% of trials in Experiment 1 and about 4% in Experiment 2) were removed from the training dataset for each experiment before data analysis.

## RESULTS

### Training performance

#### Experiment 1

Accuracy was at ceiling throughout training with passive study ($\bar{x}$ > 97% for each condition). We anticipated that the accuracy for the passive study trials during training would be nearly 100%, as participants were given the correct answer at the start of each trial. For retrieval practice, participants improved across training sessions and were significantly more accurate in Session 3 than in Sessions 1 and 2 (*p*s < 0.01, *d* = 0.73 and 0.36, respectively). Training accuracy with retrieval practice did not differ between or interact with the described connection and label‐only conditions (*p*s > 0.36). Figure 3B displays accuracy for each Experiment 1 training session and condition.

#### Experiment 2

Participants improved across training sessions for all conditions, showing higher accuracy in Sessions 2 and 3 than in Session 1 (*p*s < 0.001, *d* = 0.54 and 0.40, respectively). Accuracy did not reliably differ among conditions during the training sessions, and there were no significant interactions between training session and condition (*p*s > 0.10). Figure 3C displays the accuracy for each training session in Experiment 2.

### Final memory tests

#### Experiment 1

As shown in Figure 4, both described connections and retrieval practice improved final memory test performance. A repeated‐measures ANOVA revealed significant main effects for both described neural connections (described connection vs. label‐only: *F* (1, 24) = 7.38, *p* = 0.01, *d* = 0.54) and retrieval practice (retrieval practice vs. passive study: *F* (1, 24) = 5.47, *p* = 0.03, *d* = 0.47). There was no evidence of an interaction between factors (described neural connections × retrieval practice: *F* (1, 24) = 0.00, *p* = 0.96). Performance did not differ between the female and male students, and gender did not significantly interact with either of the training manipulations (*p*s > 0.05).

#### Experiment 2

**FIGURE 5:**
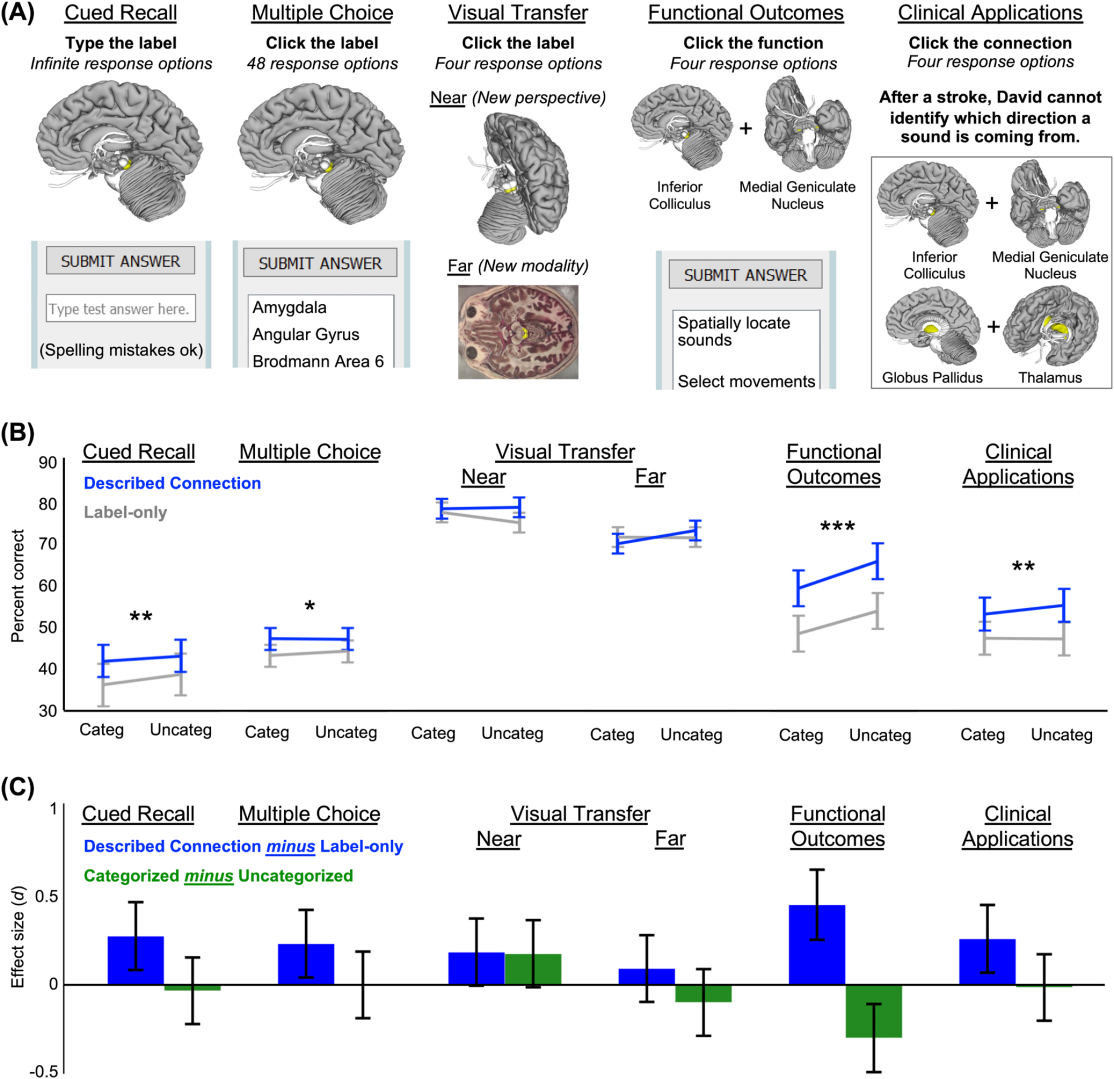
Experiment 2 long‐term memory. (A) Long‐term memory and knowledge generalization for each brain area were tested in six ways. (B) We observed significant advantages in long‐term memory associated with both the described neural connections and retrieval practice. Interactions between training manipulations were not reliable for any measure. 95% confidence intervals and significance markers reflect the main effect of described neural connections. (C) Effect sizes for the two training manipulations and each outcome measure with 95% confidence intervals for each effect size. *p < 0.05; **p < 0.01; ***p < 0.001.

Described neural connections benefited performance on most memory tests (positive blue bars in Figure 5C). Critically, this included significant main effects for both measures of structure identification: cued recall (*F* (1, 102) = 8.08, *p* = 0.005, *d* = 0.28) and multiple choice (*F* (1, 102) = 5.75, *p* = 0.02, *d* = 0.24). The significant main effect of described connections in the multiple‐choice test was a direct replication of the described neural connections effect in Experiment 1. Additionally, although described neural connections did not significantly affect performance on tests of near visual transfer (*F* (1, 102) = 3.64, *p* = 0.06, *d* = 0.19) or far visual transfer (*F* (1, 102) = 0.90, *p* = 0.35, *d* = 0.09), they benefited performance on tests of functional outcome (*F* (1, 102) = 21.78, *p* < 0.001, *d* = 0.46) and clinical application (*F* (1, 102) = 7.19, *p* = 0.009, *d* = 0.27).

Categorization had negligible effects on any of the final memory tests (green bars in Figure 5C). The main effect of categorization did not approach significance for cued recall, multiple choice, far visual transfer, or clinical applications (*p* > 0.30). It also was not reliable for near visual transfer (*F* (1, 102) = 3.29, *p* = 0.07, *d* = 0.19). In contrast, knowledge of functional outcomes was impaired for categorized structures compared with uncategorized structures (*F* (1, 102) = 9.66, *p* = 0.002, *d* = −0.31). Here, the functional outcomes of individual connections were more confusable when participants learned them within the same section of a training session and the same category (e.g., “Motor System”) than when they were intermixed across categories.

Finally, performance did not differ across institutions or genders for any of the memory tests, and there were no statistically significant interactions between the training manipulations and either institution or gender (*p*s > 0.05). Additionally, no within‐subjects interactions were statistically significant for any of the memory tests (*p*s > 0.05).

## DISCUSSION

Providing information about neural connections and their functional outcomes enhances students' ability to identify brain structures a month after training (both experiments). Training data indicated similar initial understanding across conditions, implying that observed performance differences in the final test reflect long‐term retention rather than initial learning (both experiments). This improvement in long‐term retention is comparable in effect size to that of retrieval practice (Experiment 1), and it generalizes to reasoning about cognitive and behavioral deficits in stroke patients, but it does not generalize to a new visual perspective or modality (Experiment 2). Providing information about neural connections differs from categorizing structures into labeled systems. Categorizing structures under labels like “Visual System” did not enhance long‐term memory and impaired functional outcome knowledge (Experiment 2). Below, we explore potential psychological mechanisms that explain how learning neural connections enhances students' long‐term memory of neuroanatomy. We then pose empirical questions for future research on connected neuroanatomy instruction.

### Constructing mental models through described neural connections

In each experiment, information about neural connections and their functional outcomes facilitated the memory of structure names, even though knowledge about connections is not strictly necessary to identify individual brain structures. This advantage for long‐term memory is consistent with research on the psychology of learning and theories centered on levels of processing and elaborative encoding.^79^, ^80^ Elaborative encoding in each experiment could include associations between pairs of structures,^81^ knowledge of how sets of brain areas are related to each other,^46^ and causal models of how multiple brain areas interact to produce functional outcomes.^82^ More generally, reading about neural connections and their functional outcomes may help students construct integrated mental models of the brain.

While described neural connections may help students construct new mental models of the brain, they may also assist students in integrating new information into existing mental models. Reading about neural connections and their functional outcomes can help students incorporate new information into existing cognitive frameworks. One example of this is making neuroanatomy more relevant to the real‐world understanding of brain function. In addition, it may be easier for learners to build mental models of neuroanatomy when the information to be learned shares spatial, causal, or goal‐related contexts with existing knowledge.^50^, ^83^, ^84^, ^85^ Learning about neural connections may better enable students' existing models to provide scaffolding, supporting the integration of new information into existing cognitive frameworks. For example, a student who knows that the superior colliculus supports eye movements can learn more quickly that the lateral geniculate nucleus is also part of the visual system. Likewise, students can remember the functional outcomes of neural connections and apply this knowledge to identify potentially damaged neural connections in a clinical context.

Notably, the procedure described here focuses on neural connections causing functional outcomes without specifying the causal sequence between brain regions. While reciprocal connectivity is consistent with fMRI measures of functional connectivity based on temporal correlations among brain structures,^86^ future studies could extend to causal relationships within the brain (Structure A *causes* Structure B to cause an outcome). Such specification could further help knowledge transfer to clinical applications because specific parts of a causal and temporal chain may account for neuropsychological symptoms. For example, in a clinical situation, a neurologist may need to determine the underlying cause of a patient's visual impairment. Understanding the neural connections involved in visual processing, such as the pathway from the retina to the primary visual cortex, is crucial for diagnosing and treating the condition.

### Curricular integration through described neural connections

We showed that encouraging students to build mental models based on neural connections helps them learn and retain fundamental neuroanatomy. Such integration can provide a rich contextual resource for the information being learned in the course. In introductory neuroscience classes, where functional neuroanatomy is an element of the broader course content,^87^, ^88^ the material covered throughout the course may provide particular support and incentive for emphasizing neural connections in the comprehension and retention of neuroanatomy.

### Separate effects of retrieval practice

Retrieval practice benefited memory in Experiment 1 with a similar effect size to the learning benefit provided by described neural connections. The reliable main effect of retrieval practice replicates an extensive literature on retrieval practice effects in other domains^62^, ^64^, ^65^, ^89^ and at least one other study in neuroanatomy instruction.^72^ The memory benefit of retrieval practice was additive with the learning benefit of described neural connections. The lack of interaction between these manipulations could suggest separate learning mechanisms. However, it is essential to consider the details of how described neural connections and retrieval practice were implemented.

Critically, elaborative encoding of neural connections occurred *after* retrieval practice of the structure name. While previous studies have reported that retrieval practice and elaborative encoding can interact when elaborative encoding is performed before retrieval practice,^90^, ^91^ additive effects, such as those found in Experiment 1, have been observed in research in which elaborative encoding is performed after retrieval practice.^92^, ^93^

### Encouraging knowledge transfer

Reading about neural connections led to the successful transfer of knowledge from functional information to clinical applications. Students effectively applied knowledge of functional outcomes to problem‐solving in clinical situations involving damaged neural connections. Knowledge transfer to clinical applications required inferring the functional consequences of losing a connection.

Reading about neural connections did not enhance the visual transfer necessary to recognize brain structures from different camera angles and visual contexts that include different surrounding brain structures. The lack of visual transfer observed for the near and far visual transfer tests warrants further investigation. Near and far visual transfer may be more effectively encouraged by including more varied brain imagery during training and feedback about neural connections. A strength of SMtB is the ability to examine structures from different angles and with various collections of surrounding structures.^58^, ^59^ Future studies could specifically target visual transfer by beginning each trial with the brain in a different orientation or with other surrounding structures.

### Minimal effects of categorization

Categorizing brain structures by functional system labels alone did not enhance students' memory or generalization of neuroanatomical knowledge. Generally, students learn information better when items are organized according to meaningful relationships rather than perceived as arbitrary collections.^94^, ^95^, ^96^ However, the categorization implemented in the current experiments involved system labels (e.g., “Visual System,” “Motor System”) without explicitly presenting the underlying neural connections or hierarchical relationships among structures. This limited internal organization may have rendered the categories too abstract, thereby increasing cognitive load rather than providing meaningful scaffolding for learning.^97^, ^98^ Indeed, rather than aiding memory, categorization impaired the ability to remember specific functional outcomes associated with individual neural connections. Such impairment likely arose because students struggled to effectively integrate the abstract category labels with the functional information, thereby hindering comprehension and application. Future research could explore more structured hierarchical categorizations or explicitly linking system labels with concrete neural connections to reduce cognitive load and enhance students' ability to integrate and apply their neuroanatomical knowledge.

### Insights from training data

Significant effects emerged in the final memory tests (after a 4.5‐week delay) that were not observed during the training sessions. Notably, Experiment 1 training involved two alternative multiple‐choice questions, while Experiment 2 training used four alternative multiple‐choice questions. These formats are easier and less sensitive than the 48‐alternative multiple‐choice and cued recall formats used in the final memory tests. However, both experiments demonstrated reliable improvements in accuracy across training sessions, indicating meaningful learning over time. Therefore, the pattern across training and final memory tests suggests that the benefits of the described neural connections may be specific to long‐term retention rather than initial learning.

### Limitations of the current study

Results suggest that described neural connections with functional outcomes can enhance long‐term memory of neuroanatomy. However, neural connections can be represented in multiple ways beyond verbal descriptions of function. For instance, the white matter fiber tracts that form neural connections have names, and they can be visualized using diffusion tractography,^99^ fMRI functional connectivity,^31^ anterograde and retrograde tracing,^100^ electrophysiology,^101^ and optogenetics.^102^ Likewise, mental models of systems and events are multidimensional representations with functional, spatial, temporal, causal, and other contextual features.^84^, ^85^ Additional components of neural connectivity could help students construct richly connected mental models of the brain that enhance long‐term memory.

From the perspective of Bloom's taxonomy of educational objectives,^103^, ^104^, ^105^ the training manipulations and memory tests were limited to knowledge (Level 1 of Bloom's taxonomy) and comprehension (Level 2), without extending to application or analysis (Levels 3 and 4, respectively). Specifically, the tests in Experiment 1 were at Level 1 of Bloom's taxonomy. Experiment 2 reaches Level 2 (Functional Outcomes and Clinical Applications) and possibly Level 3 (Visual Transfer) of the Blooming Anatomy Tool. The Clinical Application question falls short, as removing the first half of the sentence would still produce the correct answer. For a robust clinical application, it would be helpful to display an MRI showing a lesion in the area of the inferior colliculus and medial geniculate nucleus.

Most students at each institution were female. Each course was offered in the psychology department of the respective institution, and the ratio of male to female students in each study aligned with the gender demographics of psychology majors at each university. While we might otherwise predict a gender difference for some of the memory tests in the near transfer condition that required some degree of mental rotation,^106^ gender differences were not observed in this study.

Our findings suggest that connectivity‐based neuroanatomy instruction can be successfully integrated into introductory neuroscience courses typically taken by second‐ or third‐year undergraduates. Future studies could examine samples of more experienced students, such as those enrolled in medical schools, whose prior knowledge and cognitive strategies may differ significantly due to their focused biomedical training.^107^, ^108^ Specifically, medical students might employ distinct learning approaches even in their initial training stages, potentially influencing their ability to integrate and apply functional neural connections effectively.^34^, ^109^ Investigating these differences could help optimize instructional approaches tailored to diverse educational backgrounds and learning contexts.

## CONCLUSIONS

Neuroscience research has shifted from studying individual brain structures to exploring their connections. Incorporating information about neural connections into neuroanatomy education may facilitate a deeper understanding and longer‐lasting memory of the material. Classroom‐based findings here challenge the traditional emphasis within neuroanatomy education on individual brain structures in favor of a more integrated learning approach that reflects the brain's interconnected nature. Focusing on neural connections can improve undergraduate neuroscience education by leveraging the connectivity paradigm shift in neuroscience research.

## AUTHOR CONTRIBUTIONS

Nicholas C. Hindy, Anthony J. Bishara, and John R. Pani designed the experiments, reviewed the analyses, discussed the results, and wrote the manuscript. John R. Pani created the “Show Me the Brain!!” software platform for neuroanatomy training and final memory tests. Nicholas C. Hindy coordinated data collection and performed the analyses.

## FUNDING INFORMATION

This work was supported by the National Science Foundation's Improving Undergraduate STEM Education (IUSE) initiative (NSF Award no.: 2315440: An Online Platform for Learning Neuroanatomy from Neural Connectivity; PI: Nicholas Hindy).

## CONFLICT OF INTEREST STATEMENT

The authors declare no conflicts of interest.

## Supporting information


Table S1.
Table S2.

## Data Availability

Data and other materials for each experiment are publicly available in an Open Science Framework repository that can be accessed at https://osf.io/dnu9x.
