# Supplementary material for: Described neural connections enhance classroom learning of neuroanatomy
Source: Anat Sci Educ. 2025 Jun 7;18(7):642–56. doi: 10.1002/ase.70051 (PMC12222580; doi:10.1002/ase.70051)
Supplement: Supplementary file 1 — Table S1. Table S2. [file ASE-18-642-s001.pdf]

Supplemental Material

TABLE S1.

Brain structures and described neural connections. Each experiment included 48 brain structures sampled from four functional anatomical categories (linguistic, limbic, motor, and visual). Described neural connections were shown as text-based narrative feedback after training trials. In Experiment 1, we selected brain structures within each condition from multiple categories. In Experiment 2, brain structures were either drawn from multiple anatomical categories ("uncategorized" conditions) or a single category ("categorized" conditions).

	Brain Structure	Described Neural Connection
Linguistic	Angular Gyrus	Connects with the planum temporale to comprehend language
	Planum Temporale	Connects with the angular gyrus to comprehend language
	Brodmann Area 44	Connects with Brodmann area 45 to produce speech
	Brodmann Area 45	Connects with Brodmann area 44 to produce speech
	Middle Frontal Gyrus	Connects with Brodmann area 47 to retrieve words
	Brodmann Area 47	Connects with the middle frontal gyrus to retrieve words
	Inferior Colliculus	Connects with the medial geniculate nucleus to localize sounds
	Medial Geniculate Nucleus	Connects with the inferior colliculus to localize sounds
	Middle Temporal Gyrus	Connects with the temporal pole to store knowledge
	Temporal Pole	Connects with the middle temporal gyrus to store knowledge
	Superior Temporal Gyrus	Connects with the transverse temporal gyrus to perceive tones
	Transverse Temporal Gyrus	Connects with the superior temporal gyrus to perceive tones
Limbic	Amygdala	Connects with the stria terminalis to process emotion
	Stria Terminalis	Connects with the amygdala to process emotion
	Cingulate Gyrus	Connects with the insula to maintain motivation
	Insula	Connects with the cingulate gyrus to maintain motivation
	Fornix	Connects with the nucleus accumbens to learn rewards
	Nucleus Accumbens	Connects with the fornix to learn rewards
	Hippocampus	Connects with the parahippocampal gyrus to create memories
	Parahippocampal Gyrus	Connects with the hippocampus to create memories
	Hypothalamus	Connects with the pituitary gland to control hormones
	Pituitary Gland	Connects with the hypothalamus to control hormones
	Lateral Orbitofrontal Gyrus	Connects with the medial orbitofrontal gyrus to make decisions
	Medial Orbitofrontal Gyrus	Connects with the lateral orbitofrontal gyrus to make decisions

TABLE S1. (continued)

	Brain Structure	Described Neural Connection
Visual	Brodmann Area 17	Connects with Brodmann area 18 to see fine details
	Brodmann Area 18	Connects with Brodmann area 17 to see fine details
	Superior Colliculus	Connects with the lateral geniculate nucleus to control eye movements
	Lateral Geniculate Nucleus	Connects with the superior colliculus to control eye movements
	Optic Tract	Connects with the optic nerve to transmit visual information
	Optic Nerve	Connects with the optic tract to transmit visual information
	Superior Parietal Lobule	Connects with the precuneus to shift spatial attention
	Precuneus	Connects with the superior parietal lobule to shift spatial attention
	Superior Occipital Gyrus	Connects with the inferior occipital gyrus to perceive shapes
	Inferior Occipital Gyrus	Connects with the superior occipital gyrus to perceive shapes
	Fusiform Gyrus	Connects with the inferior temporal gyrus to recognize objects
	Inferior Temporal Gyrus	Connects with the fusiform gyrus to recognize objects
Motor	Precentral Gyrus	Connects with Brodmann area 6 to direct behavior
	Brodmann Area 6	Connects with the precentral gyrus to direct behavior
	Superior Frontal Gyrus	Connects with Brodmann area 8 to plan actions
	Brodmann Area 8	Connects with the superior frontal gyrus to plan actions
	Caudate Nucleus	Connects with the putamen to select movements
	Putamen	Connects with the caudate nucleus to select movements
	Cerebellum	Connects with the red nucleus to develop skills
	Red Nucleus	Connects with the cerebellum to develop skills
	Thalamus	Connects with the cerebral peduncles to relay commands
	Cerebral Peduncles	Connects with the thalamus to relay commands
	Subthalamic Nucleus	Connects with the globus pallidus to inhibit responses
	Globus Pallidus	Connects with the subthalamic nucleus to inhibit responses

TABLE S2.

Incorrect answer options for multiple-choice training questions. To avoid students guessing the correct answers, foils in each experiment were randomly selected from a group of 15-17 similar structures for each correct structure label. For example, if "Amygdala" is the correct answer, an incorrect option could have been "Hypothalamus." Answer options included two different structure labels for each training trial in Experiment 1 and four different labels for each trial in Experiment 2.

**Foil Group 1**	**Foil Group 2**	**Foil Group 3**
Amygdala	Brodmann Area 6	Angular Gyrus
Caudate Nucleus	Brodmann Area 8	Cingulate Gyrus
Globus Pallidus	Brodmann Area 17	Fusiform Gyrus
Hippocampus	Brodmann Area 18	Inferior Occipital Gyrus
Hypothalamus	Brodmann Area 41	Inferior Temporal Gyrus
Inferior Colliculus	Brodmann Area 42	Lateral Occipital Gyrus
Lateral Geniculate Nucleus	Brodmann Area 43	Lateral Orbitofrontal Gyrus
Mammillary Body	Brodmann Area 44	Medial Orbitofrontal Gyrus
Medial Geniculate Nucleus	Brodmann Area 45	Middle Frontal Gyrus
Nucleus Accumbens	Brodmann Area 47	Middle Temporal Gyrus
Pituitary Gland	Insula	Parahippocampal Gyrus
Putamen	Planum Temporale	Precentral Gyrus
Red Nucleus	Precuneus	Superior Frontal Gyrus
Substantia Nigra	Superior Parietal Lobule	Superior Occipital Gyrus
Subthalamic Nucleus	Temporal Pole	Superior Temporal Gyrus
Superior Colliculus		Supramarginal Gyrus
Thalamus		
